# HEALTH PROFESSIONS AND MEDICAL EDUCATION: HOW THE GSA STUDENT CHAPTER ENHANCES PROFESSIONALISM AND NETWORKING

**DOI:** 10.1093/geroni/igae098.1322

**Published:** 2024-12-31

**Authors:** Marilyn Gugliucci

**Affiliations:** University of New England College of Osteopathic Medicine, Biddeford, Maine, United States

## Abstract

The University of New England College of Osteopathic Medicine (UNE COM) has had a Geriatrics Club since 2003. This club then became recognized as an American Geriatrics Society (AGS) Student Chapter in 2006. In 2022, we also participated as a pilot GSA Student Chapter. The benefits of being a chapter of either one or two national organizations positions students for national student memberships, national resources, and networking opportunities. The AGS provides practice materials and skills information to the medical students. However, GSA provides opportunities beyond research presentations and publications, with approximately 1200 of GSA members connected to the Emerging Scholars and Professional Organization (ESPO). This applies to all health professions students and includes interprofessional opportunities. With a strong support system and mentoring system within ESPO, The GSA Student Chapter provides advancement in learning skills, attitudes and knowledge, along with an extensive network of fellow students and practitioners to support their undergraduate (pre-clinical and clinical) health professions education. This prepares students for their health professions careers by providing national organizational connections that they can rely on throughout their careers. Forming a GSA Student Chapter for health professions students, regardless of the type of health professions program will be shared and sample programs and events in palliative care, geriatrics and gerontology will be presented.

